# Complete Mitochondrial Genomes of *Araiocypris batodes* and *Tanichthys huidongensis* Provide Phylogenetic Evidence for the Reassignment of *Araiocypris* to Tanichthyidae

**DOI:** 10.3390/genes17091153

**Published:** 2026-09-20

**Authors:** Liangjie Zhao, Chunhui Liu, Shufang Gao, Junjie Wang, Chaoqun Su, Chenxi Tan, Jiahui Liu, Gaoyou Yao, Xusheng Guo, Shaoyong Jing, Tiezhu Yang, Fan Li

**Affiliations:** 1 School of Fisheries, Xinyang Agriculture and Forestry University, Xinyang 464000, China; 2 Fishery Biological Engineering Technology Research Center of Henan Province, Xinyang 464000, China; 3 Zunyi Vocational and Technical College, Zunyi 563000, China; 4 School of Life Science, South China Normal University, Guangzhou 510631, China; 5 Branch of Shanghai Science and Technology Museum, Shanghai Natural History Museum, Shanghai 200041, China

**Keywords:** *Araiocypris batodes*, Tanichthyidae, mitochondrial genome, phylogenetic analysis, comparative mitogenomics, Xenocyprididae, *Tanichthys*

## Abstract

**Background/Objectives**: The monotypic genus *Araiocypris* is currently classified in Xenocyprididae, yet its familial placement has long been controversial and lacks sufficient molecular phylogenetic support. Mitogenomic resources for the family Tanichthyidae remain limited, and the phylogenetic relationship between *Araiocypris* and *tanichthyid* species remains unexplored. This study was conducted to re-evaluate the taxonomic status of *Araiocypris* and characterize the mitogenomic evolution of Tanichthyidae. **Methods**: The complete mitochondrial genomes of *Tanichthys huidongensis* and *Araiocypris batodes* were sequenced, assembled and annotated. Comparative mitogenomic analyses were performed across seven Tanichthyidae species. A maximum-likelihood phylogenetic tree was reconstructed using concatenated sequences of 13 protein-coding genes and 2 ribosomal RNA genes to clarify the phylogenetic position of *Araiocypris*. **Results**: Based on mitochondrial phylogenomic evidence, phylogenetic reconstruction strongly supports the reassignment of *A. batodes* from Xenocyprididae to Tanichthyidae, as it nests within the Tanichthyidae clade and forms a sister group with the genus *Tanichthys*. Genetic divergence analysis confirms the validity of *Araiocypris* as a distinct genus. Tanichthyidae mitogenomes display highly conserved architecture with the typical vertebrate gene order. All protein-coding genes undergo strong purifying selection, with *cox1* showing the slowest evolutionary rate. Conserved structural domains including CSB-F, CSB-E, CSB-D and CSB1–3 were identified in the control region. **Conclusions**: This study provides solid mitogenomic evidence for the familial reclassification of *Araiocypris* and systematically reveals the evolutionary patterns of Tanichthyidae mitogenomes, offering an important reference for further taxonomic and phylogenetic studies of Cypriniformes.

## 1. Introduction

Cypriniformes is the most species-rich order of freshwater vertebrates globally, comprising more than 4000 recognized species with extraordinary ecological and morphological diversity [1]. Resolving robust phylogenetic relationships is a cornerstone for understanding the evolutionary diversification of this group and guiding evidence-based biodiversity conservation. Complete mitochondrial genomes have emerged as a robust molecular marker for resolving teleost phylogenetic relationships, owing to their high copy number, relatively fast evolutionary rate, and straightforward orthology assignment compared with nuclear loci [2,3]. For miniaturized freshwater fish lineages that often harbor cryptic species and suffer from long-standing taxonomic ambiguity, mitogenome data offer critical molecular evidence to refine taxonomic frameworks and clarify evolutionary affinities.

The family Tanichthyidae is a small cypriniform lineage containing the single genus *Tanichthys*, with native populations restricted to southern China and northern Vietnam [4]. Nine valid species are currently recognised in the genus *Tanichthys*, including seven species known from China (*Tanichthys albiventris*, *Tanichthys albonubes*, *Tanichthys flavianalis*, *Tanichthys guipingensis*, *T. huidongensis*, *Tanichthys luheensis*, *Tanichthys shenzhenensis*) and two recorded from Vietnam (*Tanichthys kuehnei*, *Tanichthys micagemmae*) [5,6,7]. Most of these species have been newly described in recent years. Meanwhile, mitochondrial genome studies of these taxa have followed, improving our understanding of the evolutionary history of Tanichthyidae [8,9]. Historically, both tanichthyid and xenocypridid lineages were placed within the broadly circumscribed family Cyprinidae, mostly assigned at subfamilial or tribal rank due to their overall morphological similarity to core cyprinid fishes. Over the past two decades, cumulative morphological and molecular phylogenetic evidence has gradually led to the recognition of Xenocyprididae and Tanichthyidae as distinct, valid families within Cypriniformes. However, the familial placement of many small, rarely collected genera from this broader clade remained unassessed and controversial during this taxonomic restructuring.

The monotypic genus *Araiocypris*, with *A. batodes* as its type species, is known from Quang Ninh Province in northern Vietnam and Fangchenggang City, Guangxi Zhuang Autonomous Region, China [10,11]. This small cyprinoid fish reaches 33.2 mm SL and is comparable in body size to species of *Tanichthys*. *Araiocypris* is characterised by a reduced cephalic sensory system, tubular anterior nostrils, a soft ventral keel, and large conical tubercles on the lower jaw [10]. Conway & Kottelat (2008) [10] did not resolve the familial placement of this genus when describing it. Even so, they made comparisons with multiple Xenocyprididae genera exhibiting a soft ventral keel or tubercles of similar arrangement on the lower jaw. This potentially accounts for Eschmeyer et al. (2016) [12] assigning the genus to Xenocyprididae, and this taxonomic treatment was later adopted by Tan & Armbrust (2018) [13].

However, as noted by Conway & Kottelat (2008) [10], the genera *Araiocypris* and *Tanichthys* share similar colour patterns, including a dark blotch at the caudal-fin base and a bright lateral stripe extending from the eye to the caudal-fin base. Furthermore, *Araiocypris*, similar to *Tanichthys*, possesses an anal-fin origin anterior to the base of the last dorsal-fin ray, a morphological trait separating it from most members of Xenocyprididae, in which the anal-fin origin is positioned posterior to the base of the last dorsal-fin ray [5,11,13].

Against this background, this study aims to reassess the taxonomic status of *Araiocypris* and determine its familial placement within Xenocyprididae, Tanichthyidae, or other cypriniform families. We newly sequenced and annotated the complete mitogenomes of *A. batodes* and *T. huidongensis*. Following the cypriniform phylogenetic framework proposed by Stout et al. [14], we selected representative species from Gobionidae, Acheilognathidae, Leuciscidae, and Xenocyprididae, together with additional *Tanichthys* species, to reconstruct phylogenetic relationships and evaluate the familial placement of *A. batodes*. We also performed comparative mitogenomic analyses to characterize the genomic structural features of Tanichthyidae. This study is expected to clarify the phylogenetic framework of Tanichthyidae and resolve the long-standing taxonomic ambiguity surrounding the genus *Araiocypris*.

## 2. Materials and Methods

### 2.1. Sample Acquisition and Treatment

**Figure 1:**
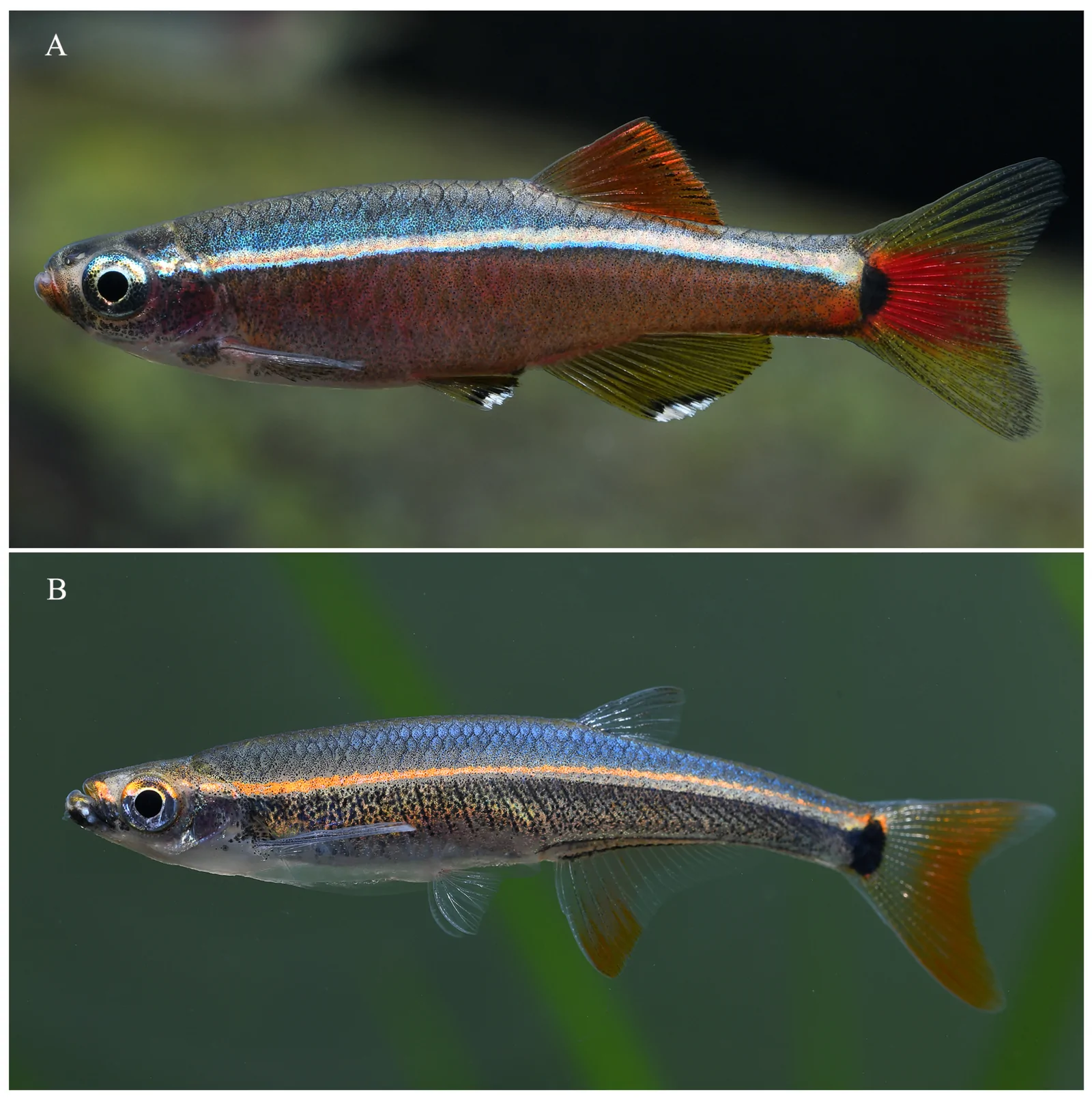
General morphology of live specimens of the two species. (**A**) *T. huidongensis*, adult specimen from the Danshui River (Xizhi River system), Huiyang, Huizhou, Guangdong; (**B**) *A. batodes*, adult specimen from the Jiangping River, Nasuo, Fangchenggang, Guangxi.

The handling of experimental animals was approved by the Animal Ethics Committee of Xinyang Agriculture and Forestry University (approval No. XYAFU-AE-20240316). Throughout the experiment, all procedures adhered to the requirements for the protection and welfare of experimental animals. A total of 6 individuals of *T. huidongensis* and 5 individuals of *A. batodes* were collected from two separate localities: the Danshui River, a headwater tributary of the Xizhi River within the Dongjiang River system of the Pearl River basin (Huiyang District, Huizhou, Guangdong Province), and the Jiangping River in Nasuo Town (Fangchenggang, Guangxi Zhuang Autonomous Region), respectively. All individuals were photographed in vivo to document morphological traits immediately after collection (Figure 1), then fixed in 95% ethanol and stored at −20 °C for long-term genomic DNA preservation. One high-quality adult specimen per species was selected for complete mitogenome sequencing. Voucher specimens of the sequenced individuals were prepared and deposited in the Ichthyological Collection of Xinyang Agriculture and Forestry University, under the accession numbers XYAFU-Mo-240723131 (*T. huidongensis*) and XYAFU-Mo-240723132 (*A. batodes*), respectively. Muscle tissues or gill filaments from *T. huidongensis* and *A. batodes* were excised and total DNA was extracted using a marine animal tissue genome DNA extraction kit (Qiagen, Beijing, China).

We downloaded the complete mitochondrial genomes of known *Tanichthys* species and some Cypriniformes from NCBI (https://www.ncbi.nlm.nih.gov/, accessed on 8 July 2024) to identify the most conserved regions. Based on these regions, eight pairs of primers were designed to amplify the complete mitogenomes of *T. huidongensis* and *A. batodes* (primer sequences are listed in Table A1 of Appendix A). The PCR conditions were set as follows: pre-denaturation at 98 °C for 1 min, followed by denaturation at 98 °C for 10 s, annealing at 56.0–58.5 °C for 30 s, and extension at 72 °C for 2 min and 30 s, with a total of 30 cycles. The reagents used included Premix Taq™ (TaKaRa, Dalian, China). The resulting amplicon sequences were then sequenced using Sanger sequencing.

### 2.2. Genomic Assembly and Annotation

We inspected raw sequencing chromatograms in BioEdit v7.2.5 to remove low-quality regions, then assembled and trimmed the overlapping amplicons into full-length mitogenomes using DNAStar v7.1 [15,16]. Both genomes were adjusted to start with the tRNA-Phe gene by aligning with published reference mitogenomes of closely related species.

The annotation of the mitochondrial genomes of both fish species was performed using the MitoAnnotator function on the online platform MitoFish (https://mitofish.aori.u-tokyo.ac.jp/, accessed on 15 September 2024) [17]. Compared to other annotation websites or software, MitoAnnotator proved to be faster and more accurate. Once the annotation was completed, the mitochondrial genomes were uploaded to the GenBank database via the Bankit program on NCBI (https://submit.ncbi.nlm.nih.gov/about/bankit/, accessed on 15 September 2024), receiving accession numbers *T. huidongensis* (PP060996) and *A. batodes* (PP060995) for the two species, respectively.

### 2.3. Phylogenetic Analyses

To clarify the familial phylogenetic position of *A. batodes* within Cypriniformes, we reconstructed a partitioned maximum likelihood (ML) phylogeny using a concatenated mitochondrial genome dataset. The final matrix covered 425 species from six cypriniform families, with Cyprinidae designated as the outgroup and the remaining five lineages (Acheilognathidae, Gobionidae, Leuciscidae, Xenocyprididae, Tanichthyidae) as ingroups. Apart from the two newly sequenced mitogenomes generated in this study, all reference sequences were retrieved from the NCBI GenBank nucleotide database. We first dereplicated the dataset and filtered out low-quality genomes with <90% completeness or incomplete annotation of core mitochondrial genes. The final dataset consisted of 13 protein-coding genes (PCGs) and two ribosomal RNA genes. To mitigate substitution saturation at synonymous sites, we excluded the third codon position of all PCGs from phylogenetic inference. For sequence alignment, we removed start and stop codons from PCGs manually, then aligned the coding sequences with MACSE v2.03, and extracted the first and second codon positions using MEGA X [18,19]. The *12S* and *16S rRNA* genes were aligned separately with MAFFT v7.490 under the L-INS-i algorithm, and ambiguously aligned regions were trimmed using the Gblocks plugin with default parameters, with gap positions retained within conserved blocks [20]. We partitioned the final alignment into three subsets: rRNA gene sequences (P1), first codon positions of PCGs (P2), and second codon positions of PCGs (P3). The three partitions were concatenated into a supermatrix using the Concatenate Sequence module of PhyloSuite v1.2.3 [21]. The best-fit substitution model for each partition was selected via the built-in ModelFinder tool, resulting in GTR + F + R7 for P1, GTR + F + R8 for P2, and GTR + F + I + R6 for P3. Partitioned ML phylogenetic inference was conducted in IQ-TREE v1.6.2, with nodal support estimated using 10,000 ultrafast bootstrap replicates [22,23]. The final phylogenetic tree was annotated and refined in the iTOL web platform (https://itol.embl.de/, accessed on 18 September 2024) and further optimized for layout in Adobe Illustrator 2021 [24].

### 2.4. Comparative Analysis of Mitochondrial Genomes in Tanichthyidae

The structure of the mitochondrial genomes of *T. huidongensis* and *A. batodes*, along with other Tanichthyidae species, was visualized using the online tool Proksee (https://proksee.ca/, accessed on 18 September 2024) [25]. RSCU calculations were performed using PhyloSuite v1.2.3, which also included statistics on the types and counts of start and stop codons in the mitochondrial protein-coding genes, as well as the usage frequency of each amino acid. The Ka/Ks ratio of each protein-coding gene was calculated using KaKs_Calculator 3.0 with the YN method, with *T. albonubes* as the reference sequence [26]. AT skew and GC skew were calculated using the formulas: AT skew = [(A − T)/(A + T)] and GC skew = [(G − C)/(G + C)]. The secondary structures of tRNA genes were predicted using the online tool tRNAscan-SE Search Server v2.0 (https://trna.ucsc.edu/tRNAscan-SE/index.html, accessed on 18 September 2024) [27]. Comparisons with closely related fish species of the *Tanichthys* genus were made to identify the control region, ETAS (Extended Terminal Associated Sequence), CD (Central Conserved Domain), and CSB (Conserved Sequence Block). All data were visualized using Origin 2022, the online tool Chiplot (https://www.chiplot.online/, accessed on 19 September 2024), and PowerPoint software, with further enhancements made in Adobe Illustrator 2021.

### 2.5. Use of Generative Artificial Intelligence

Generative artificial intelligence was used only for grammatical polishing and language optimization of the manuscript. All content has been manually reviewed and revised by the authors, who take full responsibility for the scientific content of the manuscript.

## 3. Results and Discussion

### 3.1. Phylogenetic Position and Taxonomic Reassignment of A. batodes

*A. batodes* is currently assigned to Xenocyprididae, yet its taxonomic placement lacks sufficient molecular supporting evidence. The familial placement of *A. batodes* has long been controversial: it was originally placed in the broadly defined Cyprinidae when described [10], and subsequent taxonomic revisions assigned it to Xenocyprididae [7], while its phylogenetic affinity with other cypriniform families remained untested with molecular data. In the phylogenetic study of Cypriniformes by Stout et al. [14], Tanichthyidae and Leuciscidae are recovered as sister groups, whereas Xenocyprididae forms a sister group relationship with the clade comprising Gobionidae, Acheilognathidae, Tanichthyidae and Leuciscidae.

**Figure 2:**
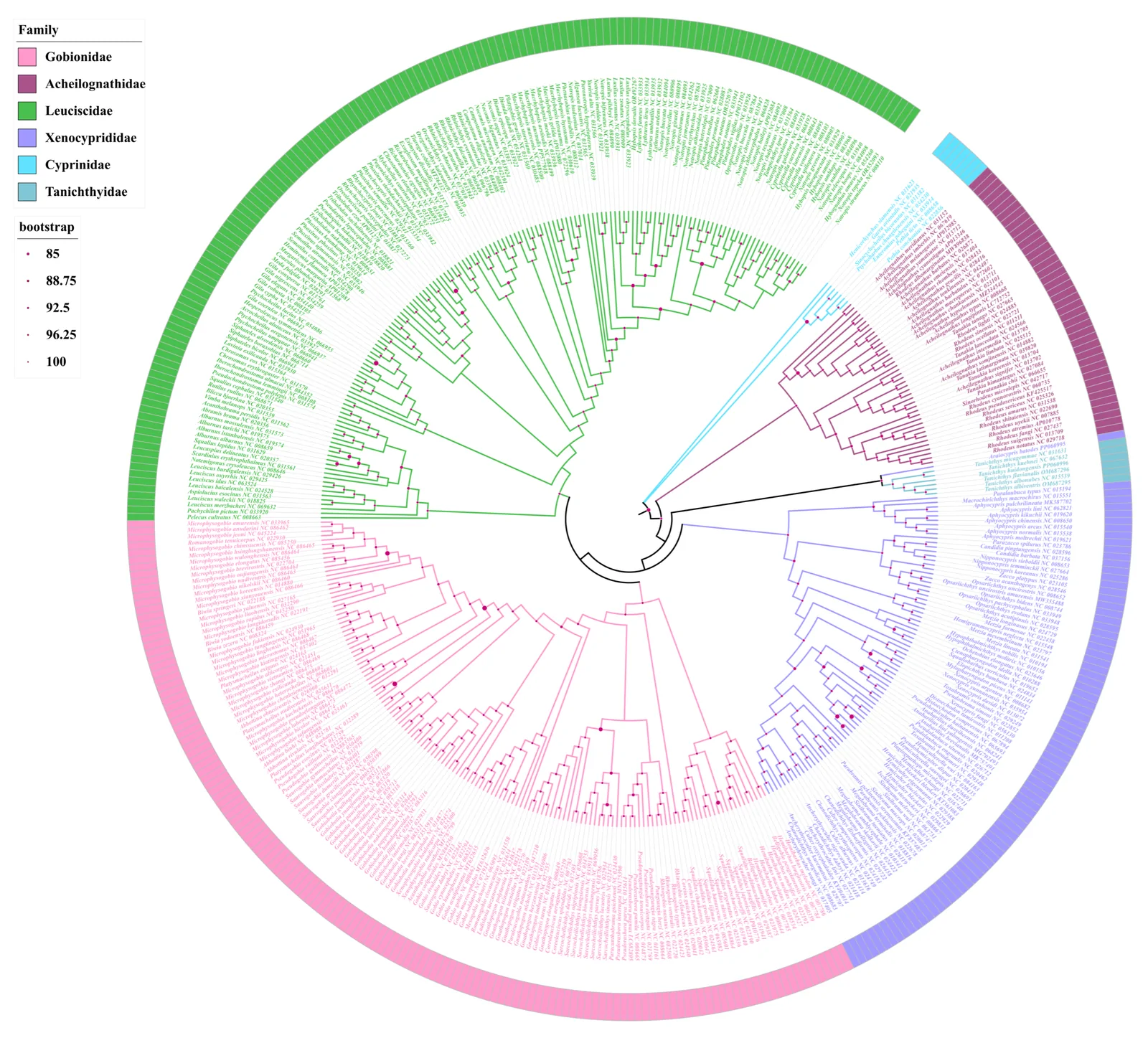
Using the maximum likelihood method, a phylogenetic tree was constructed from a dataset of 425 species across six families: Cyprinidae, Gobionidae, Acheilognathidae, Leuciscidae, Xenocyprididae, and Tanichthyidae, along with *A. batodes*. The dataset comprised rRNA genes and the first and second positions of codons from protein-coding genes in the mitochondrial genomes. The size of the red dots represents the strength of the bootstrap support values.

To re-evaluate the taxonomic status of the genus *Araiocypris*, we performed maximum-likelihood phylogenetic reconstruction including representatives of Xenocyprididae, Gobionidae, Acheilognathidae, Tanichthyidae, Leuciscidae and *A. batodes*. The resulting phylogenetic tree (Figure 2) indicates that Acheilognathidae and Tanichthyidae are sister groups with the closest phylogenetic affinity. *A. batodes* nests within the Tanichthyidae clade. Our results support the transfer of *Araiocypris* to Tanichthyidae, and indicate that it forms a sister group relationship with genus *Tanichthys*. Genetic distance and sequence similarity analyses revealed that the genetic divergence between *A. batodes* and *Tanichthys* species was generally greater than the median genetic divergence within *Tanichthys*, supporting the taxonomic validity of treating it as a distinct genus (Table 1).

From a biogeographic perspective, this phylogenetic pattern is closely linked to the geographic distribution of the two genera. As highlighted by Alvanou et al. [28] in a comparative mitogenomic study of freshwater crayfish, geographic origin of samples represents a critical factor influencing mitochondrial phylogenetic inferences, alongside formal taxonomic boundaries. In this study, *T. huidongensis* was collected from the Danshui River, a small headwater tributary of the Xizhi River in the upper Dongjiang River system of the Pearl River basin (Huiyang, Guangdong). Species of *Tanichthys* are characterized by highly restricted, allopatric distributions, with each species largely confined to isolated mountain streams across southern China, Guangxi, Hainan and northern Vietnam. Prolonged geographic isolation between independent drainage systems has been widely recognized as a major driver of lineage divergence and speciation in this genus [5,29].

In comparison, *A. batodes* has only been recorded from the Jiangping River and Beilun River basins along the Sino-Vietnamese border. Notably, the type locality of *A. batodes* (Jiangping River) lies within the broader natural distribution range of *Tanichthys*. The overlapping distributional ranges of the two genera imply a complex evolutionary history of divergence and dispersal. The phylogenetic topology recovered here is broadly consistent with this geographic distribution pattern, suggesting that allopatric differentiation driven by drainage isolation is a key evolutionary process shaping the diversity of Tanichthyidae.

We further compared our mitogenome-based phylogenetic inferences with previous studies of *Tanichthys* that employed nuclear molecular markers including *ENC1*, *RAG1* and microsatellite loci. The comparison showed that the interspecific relationships within *Tanichthys* resolved by complete mitogenomes are largely congruent with conclusions based on nuclear evidence [30].

Despite this congruence with biogeographic patterns and nuclear marker data, it should be noted that the above taxonomic inference is drawn solely from mitochondrial genome data. As a single, maternally inherited locus, the mitogenome only reflects the maternal evolutionary history of lineages and may be influenced by evolutionary processes such as introgressive hybridization and incomplete lineage sorting [31]. Further validation using nuclear genomic data is recommended to corroborate the familial placement of *Araiocypris* in future studies.

### 3.2. Comparative Mitogenomic Analysis of Tanichthyidae

#### 3.2.1. General Genome Structure and Base Composition

**Figure 3:**
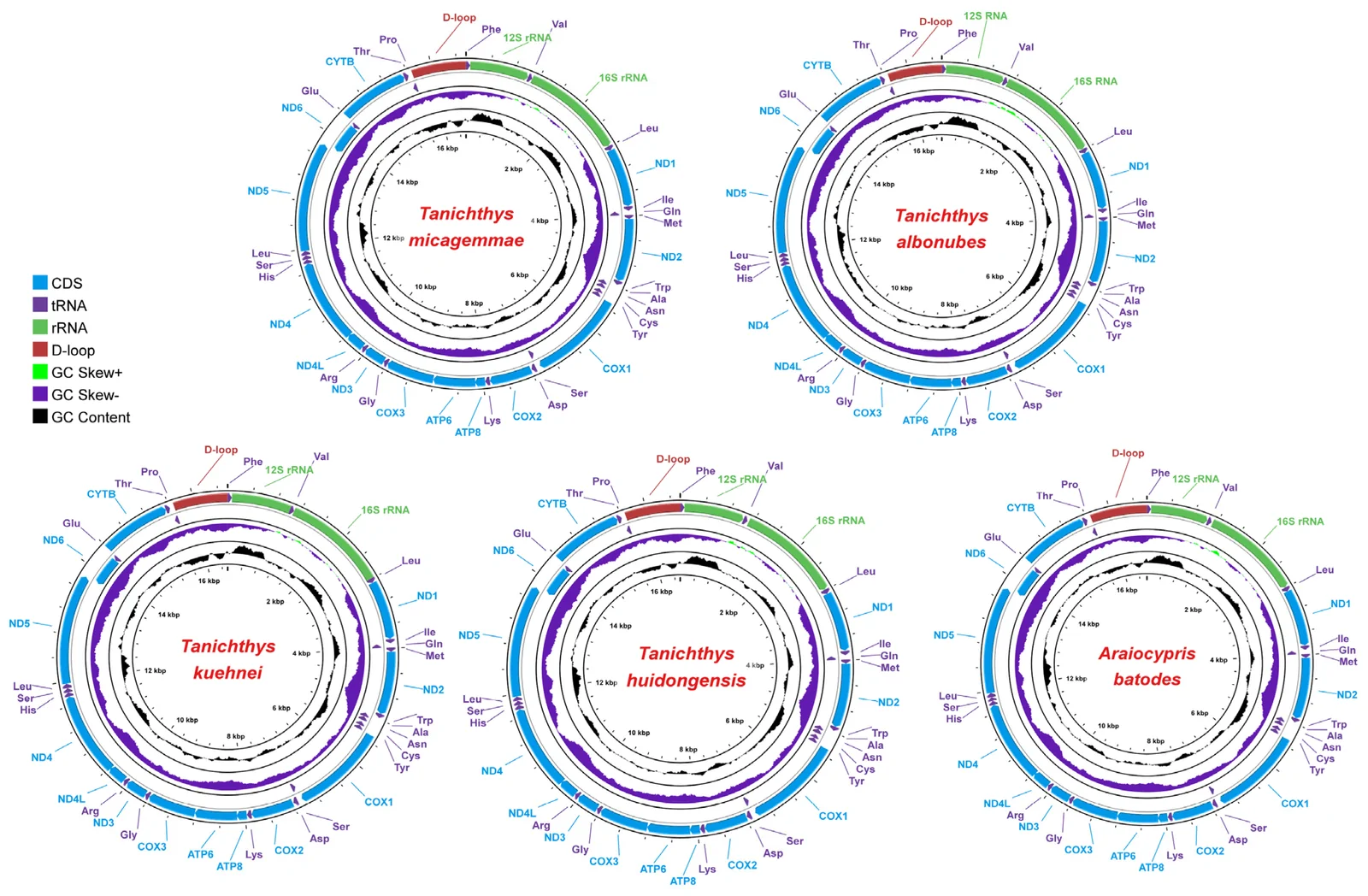
Genetic map of the complete mitochondrial genome of *T. albonubes* (NC 015539), *T. kuehnei* (NC 067632), and *T. micagemmae* (NC 031631), *T. huidongensis* (PP 060996) and *A. batodes* (PP 060995). Among these, *T. huidongensis* and *A. batodes* are the study species in this paper, while the others were obtained from GenBank (https://www.ncbi.nlm.nih.gov/nuccore/, accessed on 18 September 2024). The mitochondrial genome structures of *T. albiventris* (OM 687295) and *T. flavianalis* (OM 687296) have been previously presented in other studies; therefore, this research does not provide visualizations for them [8,9].

The known complete mitochondrial genome data for the family Tanichthyidae available on NCBI include five species: *T. albonubes* (NC 015539), *T. albiventris* (OM 687295), *T. flavianalis* (OM 687296), *T. kuehnei* (NC 067632), and *T. micagemmae* (NC 031631). We newly sequenced, assembled, and annotated the complete mitochondrial genomes of *T. huidongensis* (accession no. PP060996) and *A. batodes* (accession no. PP060995). The full annotation details of the two mitogenomes are provided in Table A2 and Table A3 of Appendix A, respectively. A comparative analysis of the mitochondrial genomes of these seven species was conducted. The mitochondrial genomes of Tanichthyidae species are structurally similar to those of classic vertebrates, comprising a closed, double-stranded circular DNA molecule that contains 13 protein-coding genes (PCGs), 22 transfer RNA genes (tRNAs), 2 ribosomal RNA genes (rRNAs), as well as 1 control region and 1 origin of light strand replication (OL). Among these, *tRNA-Gln*, *tRNA-Ala*, *tRNA-Asn*, *tRNA-Cys*, *tRNA-Ser (TGA)*, *tRNA-Glu*, *tRNA-Pro*, and the *nad6* gene are encoded on the light strand, while the remaining genes are encoded on the heavy strand. No rearrangements were detected in the mitochondrial genes (Figure 3). However, rearrangements of *tRNA-Pro* and part of the control region have been observed in species of the genus *Garra*, although most Cypriniformes exhibit the typical gene arrangement found in vertebrate mitochondrial genomes [32,33]. The length of the mitochondrial genomes ranges from 16,544 bp to 16,617 bp, with *A. batodes* having the longest length at 16,617 bp, while *T. huidongensis* has a length of 16,548 bp.

**Figure 4:**
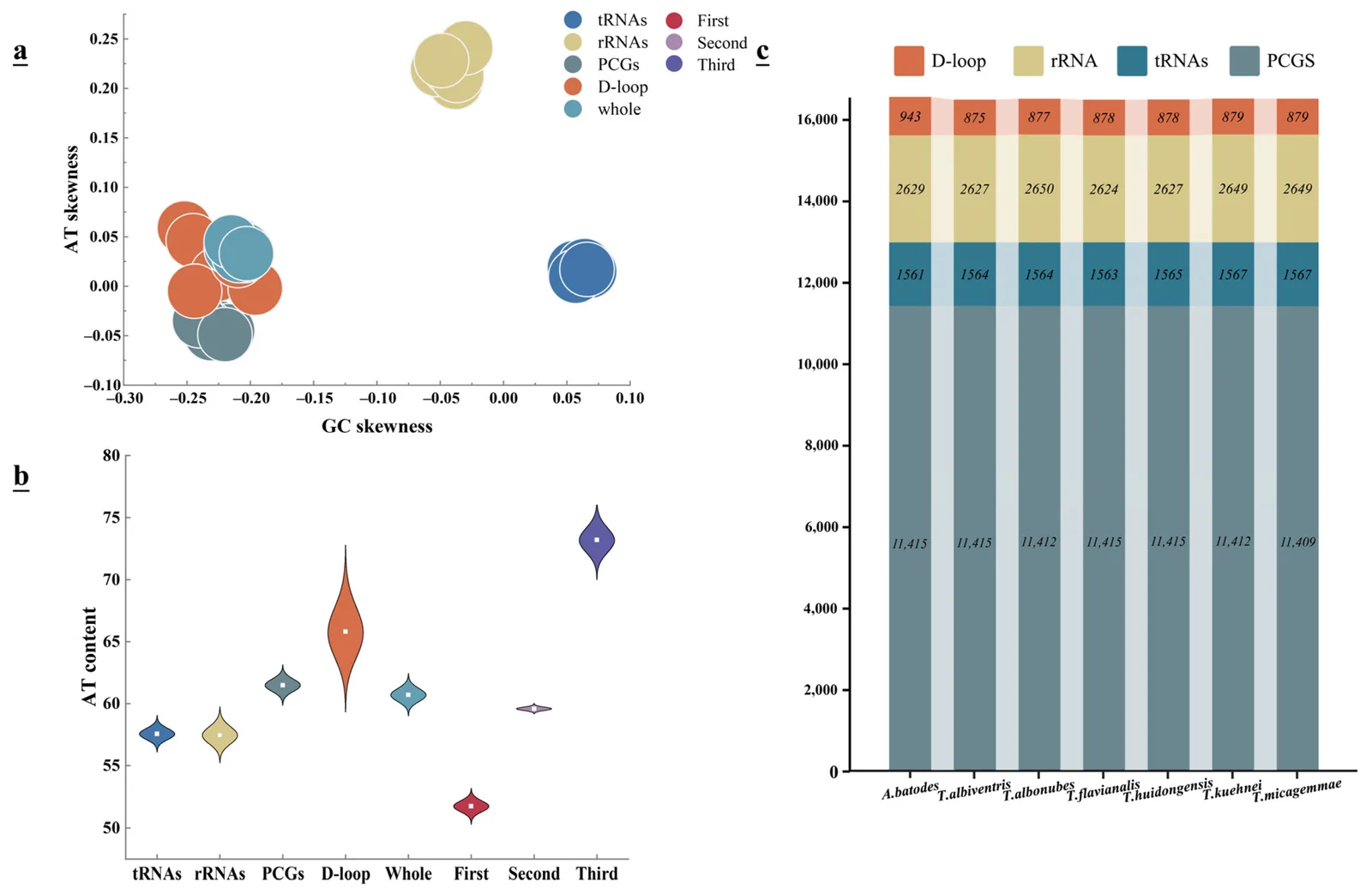
Characteristics of mitochondrial genomes and their functional components in Tanichthyidae species. (**a**) Scatter plot showing GC skewness (x-axis) and AT skewness (y-axis) across different genomic partitions: tRNAs, rRNAs, protein-coding genes (PCGs), D-loop, the whole mitogenome, and the first, second, and third codon positions. (**b**) Violin plot illustrating the A + T content distribution for the same set of genomic partitions shown in panel (**a**). (**c**) Stacked bar chart displaying the sequence length of each functional component (PCGs, tRNAs, rRNAs, and D-loop) in the mitochondrial genomes of different Tanichthyidae species.

The mitochondrial genomes of Tanichthyidae exhibit a distinct A and T base bias, similar to other Cypriniformes [34,35,36]. The content of A + T bases ranges from 59.7% to 61.2%, with a significant A and T base bias present in tRNA genes, rRNA genes, and protein-coding genes (PCGs), with a greater bias observed in the PCGs (Figure 4b). Among the A and T bases, there is a greater preference for A, while among the G and C bases, there is a greater preference for C (Figure 4a).

#### 3.2.2. Protein-Coding Genes: Base Composition, Codon Usage, and Selective Pressure

The mitochondrial genome contains 13 protein-coding genes (*nad1*, *nad2*, *cox1*, *cox2*, *atp8*, *atp6*, *cox3*, *nad3*, *nad4L*, *nad4*, *nad5*, *nad6*, *cyt b*). Except for the *nad6* gene, the other 12 genes are encoded on the heavy strand. The lengths of the 13 protein-coding genes in the mitochondrial genomes of the family Tanichthyidae range from 11,409 to 11,415 bp, with an A + T content of 60.7% to 62.1%. Among the three nucleotides in the codons of PCGs, the third position shows the greatest bias towards A and T bases, with an A + T content of 71.7% to 74.5%. Both the AT skew and GC skew are negative, indicating that the A base content is less than that of T, and the G base content is less than that of C. The AT skew value is positive, ranging from 0.031 to 0.045, indicating a higher content of A bases than T bases, while the GC skew value is negative, ranging from −0.215 to −0.203, indicating a higher content of C bases than G bases among the GC bases (Figure 4). Except for the *cox1* gene, which has a start codon of GTG, the start codons for the other 12 PCGs are all ATG. TAA and TAG are common stop codons in mitochondrial protein-coding genes. In addition to complete stop codons, there are also incomplete stop codons such as TA- and T--, which serve as stop codons for protein-coding genes in this study (Table 2). The characteristics of the start and stop codons are similar to those observed in other fish species [37,38].

**Figure 5:**
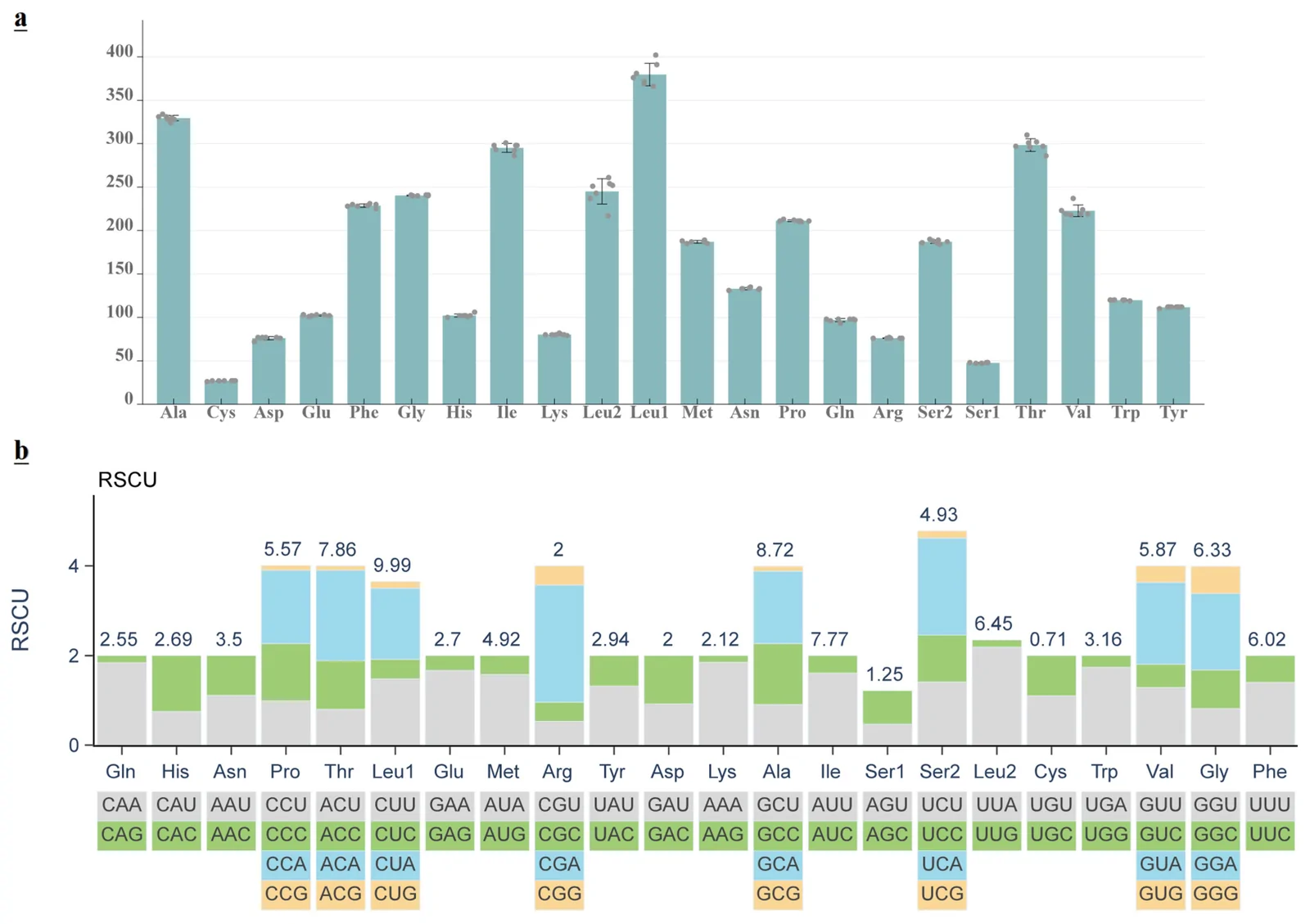
Amino acid usage (**a**) and relative synonymous codon usage (RSCU) (**b**) of the 13 protein-coding genes in the Tanichthyidae family. (**a**) The dots overlaid on each bar indicate the individual value of each species included in the analysis. (**b**) Each stacked colored block represents a synonymous codon, and the height of the block corresponds to the RSCU value of that codon.

The total number of amino acids used in the mitochondrial genomes of the seven species in the family Tanichthyidae is 3797 and 3798. Among the 22 amino acids, the preferred usage is for Leu (CUU, CUC, CUA, and CUG), Ala (GCU, GCC, GCA, and GCG), and Thr (ACU, ACC, ACA, and ACG). Cys (UGU and UGC) is used the least frequently (Figure 5a). The A + T content of the three-base codons is 50.9–52.2%, 59.4–59.8%, and 71.7–74.5%, respectively (Figure 4b). The relative codon usage (RSCU) and the base composition of the three-base codons indicate a preference for A and T bases in the third position, while among G and C bases, there is a preference for C (Figure 5b).

**Figure 6:**
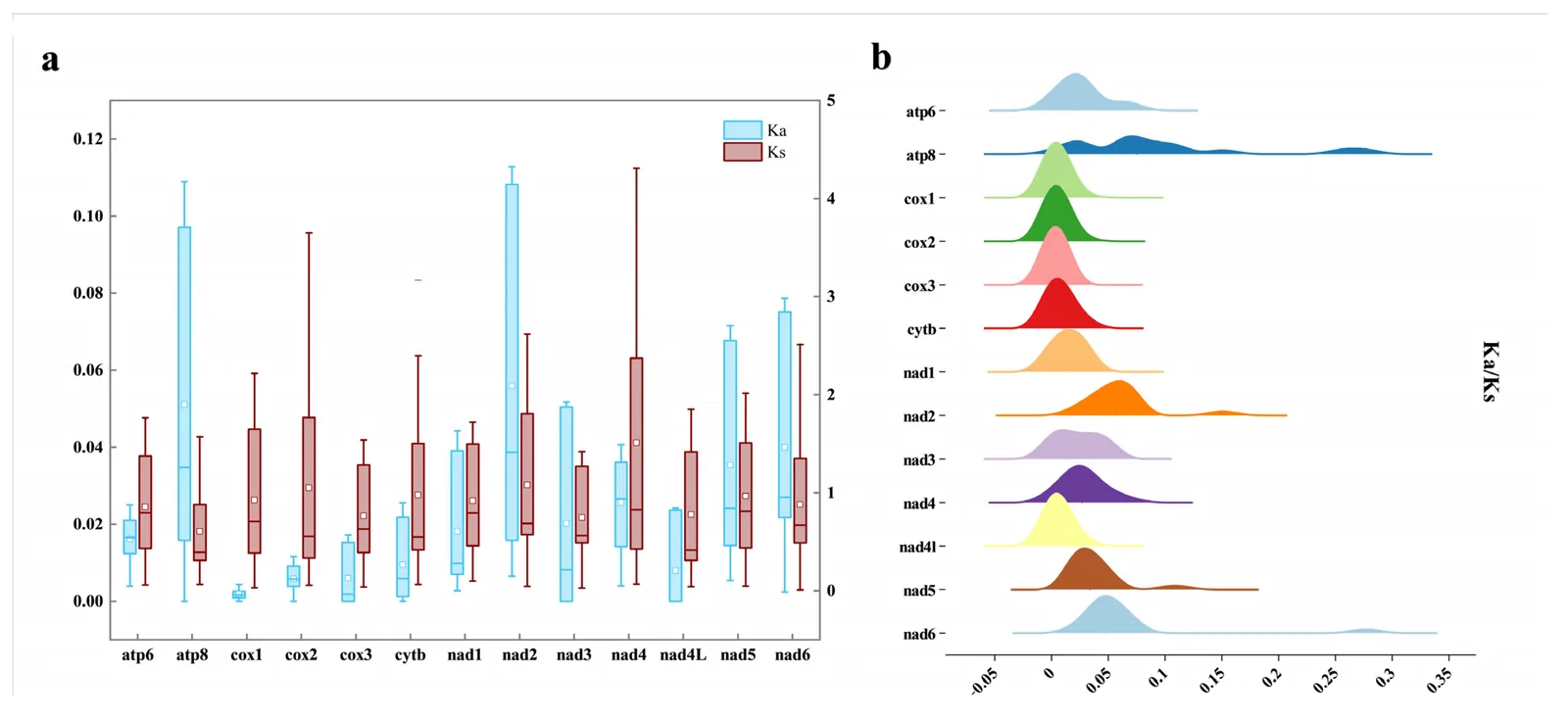
Non-synonymous substitution rate (Ka), synonymous substitution rate (Ks) and Ka/Ks ratio of the 13 mitochondrial protein-coding genes in the Tanichthyidae family. (**a**) Box plot showing the distribution of Ka (light blue) and Ks (reddish brown) across 13 protein-coding genes. (**b**) Ridge plot illustrating the distribution pattern of Ka/Ks ratios for each individual protein-coding gene.

Evolutionary rates at nonsynonymous (Ka) and synonymous (Ks) sites, as well as their ratio Ka/Ks, were calculated to quantify the strength and direction of natural selection acting on each mitochondrial protein-coding gene [39]. Across all 13 PCGs of Tanichthyidae mitogenomes, Ka/Ks ratios spanned from 0 to 0.2803, all falling well below the neutral evolution threshold of 1, indicative of pervasive purifying selection acting on the entire mitochondrial protein complement. The strength of selective constraint was not uniform across loci. Genes encoding core structural subunits of the respiratory chain complexes—*cox1*, *cox2*, *cox3* and *nad4L*—exhibited the lowest Ka/Ks values, consistent with the strongest purifying selection. Conversely, genes coding for peripheral or accessory subunits, including *atp8*, *nad2*, and *nad6*, had elevated Ka/Ks ratios, signifying relaxed selective pressure on these gene products (Figure 6b).

Ranking of nonsynonymous substitution rates further highlighted this heterogeneous evolutionary pattern: *nad2*, *atp8*, and *nad6* ranked as the fastest-evolving PCGs, whereas *cox1* evolved at the slowest rate (Figure 6a). This heterogeneous pattern of evolutionary rate is tightly linked to the functional constraints of each gene. The *cox1* gene encodes the core catalytic subunit of mitochondrial respiratory complex IV (cytochrome c oxidase), which performs the terminal step of the electron transport chain and is subject to extremely strong functional constraints. This explains its strongest purifying selection and slowest evolutionary rate, and also underpins its widespread application as a standard DNA barcode marker for fish species identification [40]. In contrast, *nad2* encodes a peripheral subunit of respiratory complex I (NADH dehydrogenase), with relatively weaker functional constraints. It therefore accumulates more nonsynonymous substitutions and exhibits a faster evolutionary rate. The *nad2* gene exhibits a relatively fast evolutionary rate among the 13 PCGs, which aligns with findings from studies on mitochondrial genomes in other fish species [41,42]. This variation in selective pressure across genes may be associated with the long-term adaptive evolution of Tanichthyidae species to their specialized stream-dwelling, miniaturized ecological niche.

#### 3.2.3. Transfer RNA and Ribosomal RNA Genes

The mitochondrial genome of Tanichthyidae contains the classic 22 tRNA genes found in vertebrate mitochondrial genomes, with a total length ranging from 1561 bp to 1567 bp. The length of individual tRNA gene sequences varies between 67 bp and 76 bp. The A + T content is between 56.8% and 58%, with a positive AT skew indicating a higher content of A bases than T bases. The GC skew value is also positive, indicating that the G base content exceeds that of the C base, which is contrary to the GC content situation in the overall mitochondrial genome (Figure 4).

**Figure 7:**
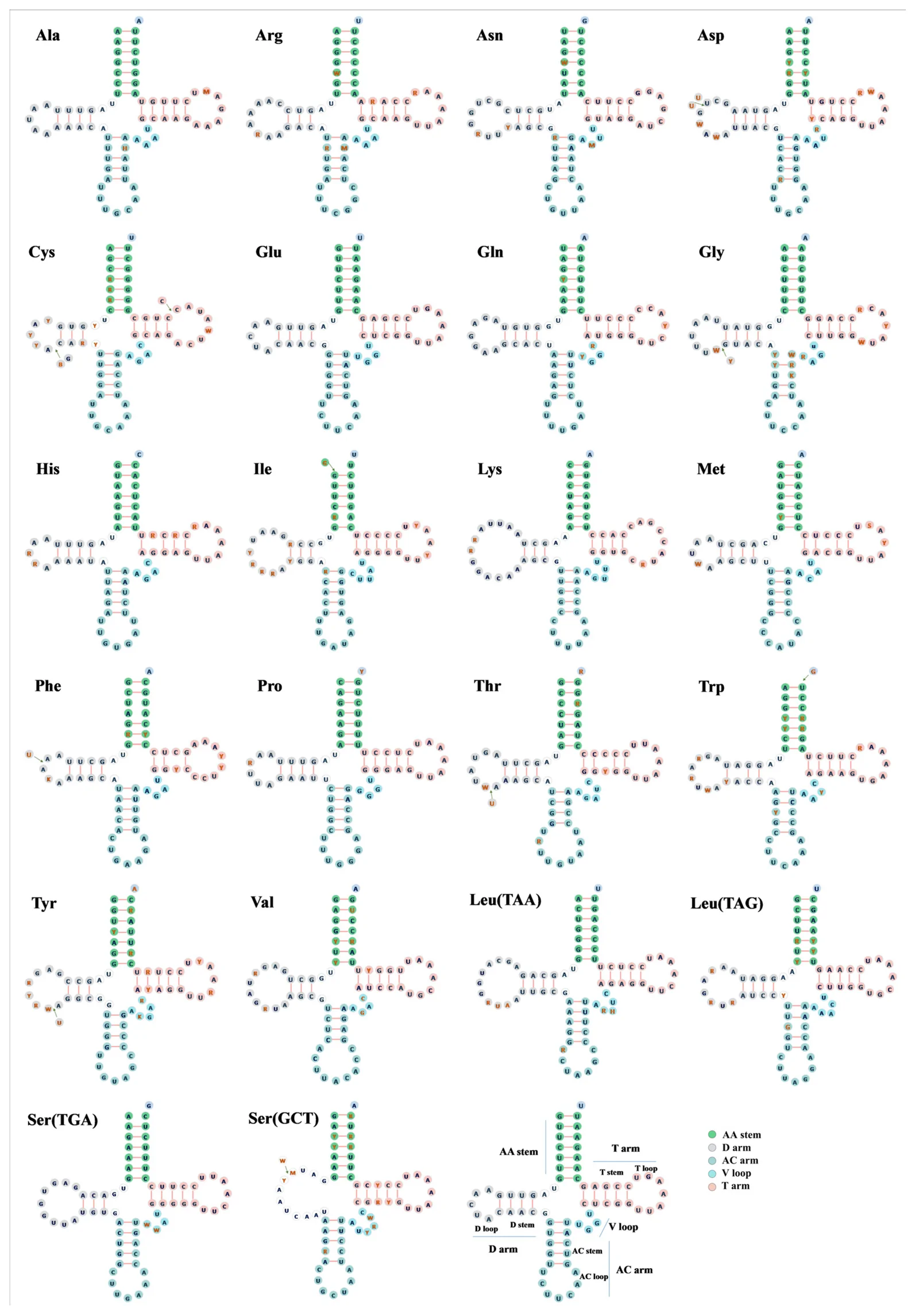
Secondary structures of tRNAs in the mitochondrial genomes of Tanichthyidae. The four arms and one loop of the tRNA cloverleaf structure (D arm, amino acid acceptor stem, anticodon arm, T arm, and variable loop) are differentiated by color coding. Base composition variations at homologous positions across species are highlighted in red; degenerate bases are labeled according to IUB standard codes: Y (C, T), W (A, T), R (A, G), K (G, T), M (A, C), H (A, C, T), and B (G, T, C) (Cornish-Bowden, 1985). Base insertion events are indicated by arrows.

Compared to other regions of the mitochondrial genome, tRNA gene sequences are relatively conserved. Their secondary structure is cloverleaf-shaped, comprising the amino acid (AA) stem, dihydrouridine (D) arm, anticodon (AC) arm, thymidine (T) arm, and variable (V) loop. In this study, all predicted tRNA secondary structures, except for *tRNA-Ser (GCT)*, which lacks a D stem, exhibit the typical cloverleaf structure. The absence of a D stem in *tRNA-Ser (GCT)* is common among fish, and similar deficiencies in D stem or T stem structures have been observed in other organisms as well [43,44,45]. The base composition of the tRNA gene sequences in the mitochondrial genomes of Tanichthyidae species is quite similar, with only *tRNA-Glu* exhibiting identical base composition. In the cloverleaf structure, the bases in the stem regions are generally more conserved, though some mismatches are present in certain tRNAs, such as U-C, A-C, U-U, A-A, and C-C mismatches. Notably, the A-C mismatch frequently occurs in *tRNA-Arg*, *tRNA-Asp*, *tRNA-His*, and *tRNA-Ile*, and is distributed across all species. The lengths of the stem structures for the 22 tRNAs are relatively fixed, characterized by an AA stem of 7 bp, a T stem of 4–5 bp, an AC stem of 4–5 bp, and a D stem of 3–4 bp (Figure 7). Variations in the number and composition of bases in the D loop and T loop are observed both within the same tRNA among different species and among different tRNAs within the same species [46]. Moreover, differences in base composition and quantity between species are primarily reflected in the loop structures.

The mitochondrial genome contains two rRNA genes: the *12S rRNA* gene and the *16S rRNA* gene, both encoded on the heavy strand, situated between the *tRNA-Phe*, *tRNA-Val*, and *tRNA-Leu (UAA)* genes. The total length of the *12S rRNA* and *16S rRNA* genes ranges from 2624 bp to 2650 bp, with an AT content between 55.9% and 57.8%. The AT skew is positive, while the GC skew is negative, indicating that the contents of A and C bases are greater than those of T and G bases.

#### 3.2.4. Non-Coding Regions and Structural Features of the Control Region

In the mitochondrial genome, the non-coding regions include the control region and the origin of replication for the light strand. The origin of light strand replication is characterized by a stem-loop secondary structure, located between the *tRNA-Cys* and *tRNA-Asn* genes, with a length of 31–32 bp. The mitochondrial control region is part of the non-coding regions of the mitochondrial genome and evolves at a relatively fast rate compared to other regions [47]. The control region is located between the *tRNA-Pro* and *tRNA-Phe* genes in the mitochondrial genome. The control region of the Tanichthyidae mitochondrial genome ranges in length from 875 to 943 bp, accounting for approximately 5.3% to 5.6% of the total genome length. The AT content is between 61.9% and 67.5%, which is higher than that of the entire mitochondrial genome. The GC bias favors C bases, and the AT skew is close to zero, indicating nearly no preference for A or T bases (Figure 4).

The control regions of all seven tanichthyid mitogenomes exhibited the typical tripartite structure of fishes, consisting of a variable ETAS domain, a central conserved domain (CD), and a conserved sequence block (CSB) region. We identified three conserved blocks (CSB-F, CSB-E, CSB-D) within the CD, as well as CSB1, CSB2 and CSB3 in the downstream CSB region, while CSB-C, CSB-B and CSB-A were not detectable in any of the species.

By comparing the control region of the Tanichthyidae mitochondrial genome with those of closely related species, it was found that this control region contains CSB-F, CSB-E, CSB-D, as well as CSB1, CSB2, and CSB3 regions [48]. Among the species compared, CSB-F, CSB-E, CSB2, and CSB3 regions are relatively conserved.

**Figure 8:**
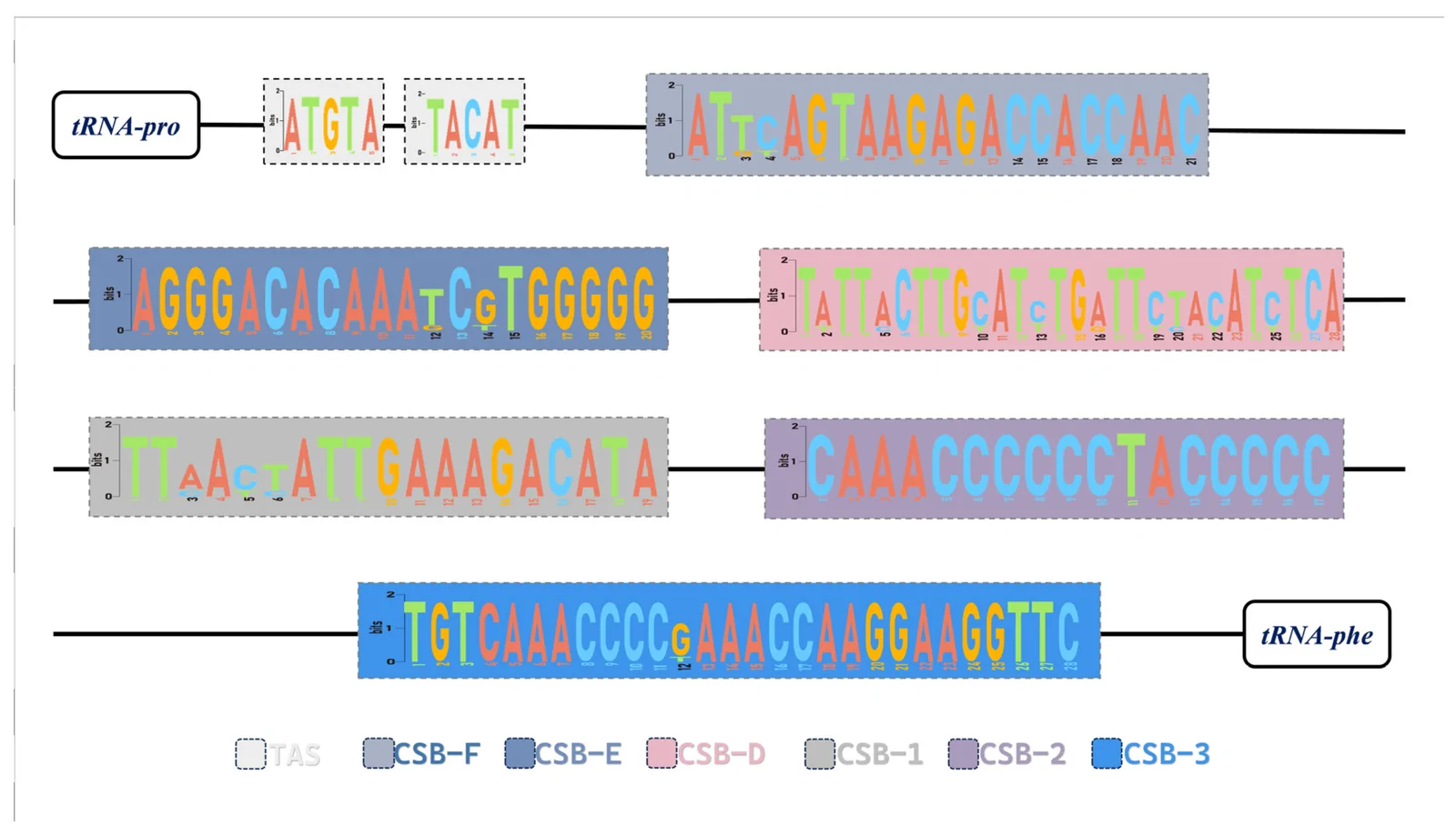
The horizontal line represents the control region of the mitochondrial genomes of Tanichthyidae, while the sequence logo illustrates the base composition of each region. The length of the spacing between the horizontal lines does not correlate with the quantity of bases.

The terminal associated sequence is the most variable region within the control region, with the CSB-F region serving as a boundary. This region has a length of 198 bp, while the termination control region of *A. batodes* is relatively longer at 251 bp. The key sequences identified are ACAT and its reverse-complementary sequence ATGT [49,50,51]. In Tanichthyidae species, the sequences ACAT and its reverse-complementary sequence ATGT range from 1 to 3 copies, and they may form a hairpin structure. Three species (*T. kuehnei*, *T. micagemmae*, *T. flavianalis*) contain a pair of complementary sequences: 5′-ATGTATTAT-3′ and 5′-TACATAA/GTA-3′ (Figure 8). All species possess the 5′-ATGTATTAT-3′ sequence (the underlined portion represents a shared sequence among seven fish species), but there are variations in the quantity and base composition of their complementary sequences.

The central conserved domain identified three regions: CSB-F, CSB-E, and CSB-D, while the regions CSB-C, CSB-B, and CSB-A could not be recognized. Based on comparisons with other species, the CSB-F region shows a significant proportion of AC bases, typically beginning with the sequence 5′-ATGTA-3′ [52]. The key sequence of the CSB-F region in *A. batodes* is quite similar to that of the reference species, with the sequence being 5′-ATGTAGTAAGAGACCACCAAC-3′, while other species have the CSB-F key sequence as 5′-TCAGTAAGAGACCACCAAC-3′. The CSB-E region is relatively conserved, with the identified key sequence being 5′-AGGGACACAAAT/GCG/T TGGGGG-3′. The GTGGG-box is present and relatively conserved in the mitochondrial genomes of most fish species [53,54]. Based on the key sequence of the CSB-D region, 5′-TATT--CTTG--ATCTG-3′, from the mitochondrial genome control region of *Rhodeus ocellatus*, the position of the CSB-D region was identified [55]. However, the nucleotide composition varies among species, with the more conserved region being 5′-CTTG-3′ (Figure 8).

The CSB1 region is associated with the origin of replication of mitochondrial DNA, making the conserved sequence region the most critical part of the entire control region. The key sequence for identifying CSB1 is 5′-GACATA-3′, which is found in the control region of most fish mitochondrial genomes. In Tanichthyidae fish, a shared sequence among several species is 5′-ATTGAAAGACATA-3′. Following the CSB-1 region, there is a poly-C sequence, 5′-G/TATGCCCCCC-3′, which is also observed in other fish species. The sequence of the CSB-2 region is the most conserved compared to other regions, consisting of 5′-CAAACCCCCCTACCCCC-3′. The CSB-3 region features a longer conserved sequence found in several species, with the sequence being 5′-TGTCAAACCCC G/T AAACCAAGGAAGGTTC-3′. After the CSB-3 region, there are 1–2 conserved sequences of 5′-CTAAAAAA-3′ (Figure 8).

## 4. Conclusions

In this study, we newly determined and annotated the complete mitochondrial genomes of *T. huidongensis* and *A. batodes*, filling gaps in mitogenomic resources for the understudied family Tanichthyidae.

Based on mitochondrial phylogenomic evidence, phylogenomic evidence consistently supports that *A. batodes* should be formally transferred from Xenocyprididae to Tanichthyidae. Within Tanichthyidae, *Araiocypris* forms a distinct sister lineage to the genus *Tanichthys*, and the genetic divergence between the two genera reaches the intergeneric level, corroborating the taxonomic validity of *Araiocypris* as an independent genus. This finding resolves the long-standing controversy over the familial assignment of *Araiocypris* and expands the known generic diversity of Tanichthyidae.

Comparative genomic analyses indicate that Tanichthyidae mitogenomes have undergone conservative evolution in genome architecture, base composition and selection pressure. The highly conserved gene arrangement and strong purifying selection on protein-coding genes reflect the evolutionary constraints on mitochondrial function in this group, while the variable terminal associated region in the control region provides potential molecular markers for population genetic and species identification studies of Tanichthyidae.

Collectively, this study establishes a robust mitogenome-based phylogenetic framework for Tanichthyidae and revises the taxonomic position of *Araiocypris*. Further studies integrating nuclear genomic data and morphological traits will not only help to comprehensively elucidate the diversification history and biogeographic patterns of Tanichthyidae within Cypriniformes, but also provide independent validation of the familial reassignment of *Araiocypris* proposed here.

## Figures and Tables

**Table 1:** Genetic distances (below diagonal) and sequence similarities (above diagonal) of mitochondrial sequences (excluding D-loop region) for Tanichthyidae species.

Species	*T. albonubes*	*T. albiventris*	*T. flavianalis*	*T. kuehnei*	*T. micagemmae*	*T. huidongensis*	*A. batodes*
*T. albonubes*		94.68%	94.17%	90.03%	90.17%	93.13%	83.75%
*T. albiventris*	0.0555		95.03%	90.39%	90.45%	93.46%	83.98%
*T. flavianalis*	0.0612	0.0516		90.26%	90.24%	93.35%	83.87%
*T. kuehnei*	0.1087	0.1046	0.1061		98.84%	90.50%	83.99%
*T. micagemmae*	0.1071	0.1038	0.1063	0.0118		90.55%	83.99%
*T. huidongensis*	0.0727	0.0690	0.0701	0.1033	0.1027		84.24%
*A. batodes*	0.1947	0.1913	0.1929	0.1917	0.1916	0.1877	

**Table 2:** Start and stop codons of protein-coding genes in the mitochondrial genome of the Tanichthyidae family.

Genes	*T. albonubes*	*T. micagemmae*	*T. kuehnei*	*T. albiventris*	*T. flavianalis*	*A. batodes*	*T. huidongensis*
*atp6*	TA/ATG	TA/ATG	TAA/ATG	TAA/ATG	TAA/ATG	TAA/ATG	TAA/ATG
*atp8*	TAA/ATG	TAA/ATG	TAA/ATG	TAA/ATG	TAA/ATG	TAA/ATG	TAA/ATG
*cox1*	TAA/GTG	TAA/GTG	TAA/GTG	TAA/GTG	TAA/GTG	TAA/GTG	TAA/GTG
*cox2*	T/ATG	T/ATG	T/ATG	T/ATG	T/ATG	T/ATG	T/ATG
*cox3*	T/ATG	T/ATG	T/ATG	TA/ATG	TA/ATG	TA/ATG	TA/ATG
*cytb*	T/ATG	T/ATG	T/ATG	T/ATG	T/ATG	T/ATG	T/ATG
*nad1*	TAG/ATG	TAG/ATG	TAG/ATG	TAG/ATG	TAG/ATG	TAA/ATG	TAG/ATG
*nad2*	T/ATG	T/ATG	T/ATG	T/ATG	T/ATG	T/ATG	T/ATG
*nad3*	T/ATG	T/ATG	T/ATG	T/ATG	T/ATG	T/ATG	T/ATG
*nad4*	T/ATG	TA/ATG	TA/ATG	TA/ATG	TA/ATG	TA/ATG	TA/ATG
*nad4L*	TAA/ATG	TAA/ATG	TAA/ATG	TAA/ATG	TAA/ATG	TAA/ATG	TAA/ATG
*nad5*	TAA/ATG	TAA/ATG	TAA/ATG	TAA/ATG	TAA/ATG	TAA/ATG	TAA/ATG
*nad6*	TAA/ATG	TAG/ATG	TAG/ATG	TAA/ATG	TAA/ATG	TAA/ATG	TAA/ATG

## Data Availability

The complete mitochondrial genome sequences newly generated in this study have been deposited in the NCBI GenBank database. The genome of *A. batodes* is available under accession number PP060995, and the genome of *T*. *huidongensis* is available under accession number PP060996. All data are publicly and freely accessible via the NCBI Nucleotide database (https://www.ncbi.nlm.nih.gov/nucleotide/, accessed on 18 September 2024).

## Abbreviations

The following abbreviations are used in this manuscript:

CD	Central conserved domain
CSB	Conserved sequence block
ETAS	Extended terminal associated sequence
Ka	Nonsynonymous substitution rate
Ks	Synonymous substitution rate
OL	Origin of light-strand replication
PCGs	Protein-coding genes
RSCU	Relative synonymous codon usage
rRNAs	Ribosomal RNAs
tRNAs	Transfer RNAs

## Appendix A

**Table A1:** Details of PCR Primers Used for Mitogenome Amplification of *A. batodes* and *T. huidongensis*.

Primer Name	Forward Primer (5′-3′)	Reverse Primer (5′-3′)	Amplicon Length (bp)	Annealing Temperature (°C)
TA-MT-1	CCCCTAGAGGAGCCTGTTCT	GCAATTAGCCATGCTCTGCC	2199	58.5
TA-MT-2	GCAGCCGCTATTAAGGGTTC	GGCTAAAGGTCATGCTGGTG	864	57.5
TA-MT-3	GCTACCCCCATTCTTGCACT	TTCACGTTTGGCAGCGAAAG	3829	56.5
TA-MT-4	GGTCTAGCCGGAATACCACG	AACCCTGTGGCCACAAAGAA	2628	56.0
TA-MT-5	CGGCTTAGCAATCTGGTTCC	AGTAGGTGTTCTCGGGTGTG	2802	58.5
TA-MT-6	TCTTGCTCGAGGGCTACAAA	GTTTTTGCTTATTCGGCGGC	2721	56.5
TA-MT-7	GACTTGAAGTCTCAGGCCCT	CGGCATGTGGGGTTAATGAG	2428	58.0
TA-MT-8	ATGAATTGGAGGAATACCCG	TACGCTACACCTCGACCTGA	1922	56.0

**Table A2:** Detailed Annotation of the Complete Mitochondrial Genome of *T. huidongensis* (accession no. PP060996).

Gene	Location		Strand	Gene Length (bp)	Intergenic Spacer (bp)	Gene Overlap (bp)	Codons	
	From	To					Start	Stop
*tRNA-Phe*	1	69	H	69				
*12S rRNA*	70	1028	H	959	2			
*tRNA-Val*	1031	1102	H	72	20			
*16S rRNA*	1123	2790	H	1668				
*tRNA-Leu(taa)*	2791	2866	H	76	1			
*nad1*	2868	3842	H	975	4		ATG	TAG
*tRNA-Ile*	3847	3918	H	72		2		
*tRNA-Gln*	3917	3987	L	71	1			
*tRNA-Met*	3989	4057	H	69				
*nad2*	4058	5104	H	1047		2	ATG	T-
*tRNA-Trp*	5103	5174	H	72	1			
*tRNA-Ala*	5176	5244	L	69	1			
*tRNA-Asn*	5246	5318	L	73				
*OL*	5319	5348	L	30				
*tRNA-Cys*	5349	5415	L	67	1			
*tRNA-Tyr*	5417	5487	L	71	1			
*cox1*	5489	7039	H	1551			GTG	TAA
*tRNA-Ser(tga)*	7040	7110	L	71	3			
*tRNA-Asp*	7114	7187	H	74	6			
*cox2*	7194	7884	H	691			ATG	T-
*tRNA-Lys*	7885	7960	H	76	1			
*atp8*	7962	8126	H	165		7	ATG	TAA
*atp6*	8120	8803	H	684		1	ATG	TAA
*cox3*	8803	9587	H	785		1	ATG	TA-
*tRNA-Gly*	9587	9657	H	71				
*nad3*	9658	10,008	H	351		2	ATG	T
*tRNA-Arg*	10,007	10,076	H	70				
*nad4l*	10,077	10,373	H	297		7	ATG	TAA
*nad4*	10,367	11,749	H	1383		1	ATG	TA
*tRNA-His*	11,749	11,817	H	69				
*tRNA-Ser(gct)*	11,818	11,886	H	69	1			
*tRNA-Leu(tag)*	11,888	11,960	H	73				
*nad5*	11,961	13,796	H	1836		4	ATG	TAA
*nad6*	13,793	14,314	L	522			ATG	TAA
*tRNA-Glu*	14,315	14,383	L	69	4			
*cyt b*	14,388	15,528	H	1141			ATG	T-
*tRNA-Thr*	15,529	15,600	H	72		1		
*tRNA-Pro*	15,600	15,669	L	70				
*D-loop*	15,670	16,548	H	879				

**Table A3:** Detailed Annotation of the Complete Mitochondrial Genome of *A. batodes* (accession no. PP060995).

Gene	Location		Strand	Gene Length (bp)	Intergenic Spacer (bp)	Gene Overlap (bp)	Codons	
	From	To					Start	Stop
*tRNA-Phe*	1	69	H	69				
*12S rRNA*	70	1033	H	964	2			
*tRNA-Val*	1036	1107	H	72	19			
*16S rRNA*	1127	2791	H	1665	1			
*tRNA-Leu(taa)*	2793	2868	H	76				
*nad1*	2869	3843	H	975	5		ATG	TAA
*tRNA-Ile*	3849	3919	H	71		2		
*tRNA-Gln*	3918	3988	L	71	1			
*tRNA-Met*	3990	4058	H	69				
*nad2*	4059	5105	H	1047		2	ATG	T-
*tRNA-Trp*	5104	5175	H	72	1			
*tRNA-Ala*	5177	5245	L	69	1			
*tRNA-Asn*	5247	5319	L	73				
*OL*	5320	5350	L	31				
*tRNA-Cys*	5351	5417	L	67	1			
*tRNA-Tyr*	5419	5488	L	70	1			
*cox1*	5490	7040	H	1551			GTG	TAA
*tRNA-Ser(tga)*	7041	7111	L	71	3			
*tRNA-Asp*	7115	7186	H	72	11			
*cox2*	7198	7888	H	691			ATG	T-
*tRNA-Lys*	7889	7964	H	76	1			
*atp8*	7966	8130	H	165		7	ATG	TAA
*atp6*	8124	8807	H	684		1	ATG	TAA
*cox3*	8807	9591	H	785		1	ATG	TA-
*tRNA-Gly*	9591	9662	H	72				
*nad3*	9663	10,013	H	351		2	ATG	T-
*tRNA-Arg*	10,012	10,081	H	70				
*nad4l*	10,082	10,378	H	297		7	ATG	TAA
*nad4*	10,372	11,754	H	1383		1	ATG	TA-
*tRNA-His*	11,754	11,822	H	69				
*tRNA-Ser(gct)*	11,823	11,890	H	68	1			
*tRNA-Leu(tag)*	11,892	11,964	H	73				
*nad5*	11,965	13,800	H	1836		4	ATG	TAA
*nad6*	13,797	14,318	L	522			ATG	TAA
*tRNA-Glu*	14,319	14,387	L	69	4			
*cyt b*	14,392	15,532	H	1141			ATG	T-
*tRNA-Thr*	15,533	15,604	H	72		1		
*tRNA-Pro*	15,604	15,673	L	70				
*D-loop*	15,674	16,617	H	944				
